# Construction of a high-density integrated genetic linkage map of rubber tree (*Hevea brasiliensis*) using genotyping-by-sequencing (GBS)

**DOI:** 10.3389/fpls.2015.00367

**Published:** 2015-05-27

**Authors:** Wirulda Pootakham, Panthita Ruang-Areerate, Nukoon Jomchai, Chutima Sonthirod, Duangjai Sangsrakru, Thippawan Yoocha, Kanikar Theerawattanasuk, Kanlaya Nirapathpongporn, Phayao Romruensukharom, Somvong Tragoonrung, Sithichoke Tangphatsornruang

**Affiliations:** ^1^National Center for Genetic Engineering and Biotechnology (BIOTEC), National Science and Technology Development AgencyPathum Thani, Thailand; ^2^Department of Agriculture, Rubber Research Institute of Thailand, Ministry of Agriculture and CooperativesBangkok, Thailand

**Keywords:** genetic linkage map, rubber tree, genotyping-by-sequencing (GBS), map integration, SNP genotyping, single nucleotide polymorphism (SNP), *Hevea brasiliensis*

## Abstract

Construction of linkage maps is crucial for genetic studies and marker-assisted breeding programs. Recent advances in next generation sequencing technologies allow for the generation of high-density linkage maps, especially in non-model species lacking extensive genomic resources. Here, we constructed a high-density integrated genetic linkage map of rubber tree (*Hevea brasiliensis*), the sole commercial producer of high-quality natural rubber. We applied a genotyping-by-sequencing (GBS) technique to simultaneously discover and genotype single nucleotide polymorphism (SNP) markers in two rubber tree populations. A total of 21,353 single nucleotide substitutions were identified, 55% of which represented transition events. GBS-based genetic maps of populations P and C comprised 1704 and 1719 markers and encompassed 2041 cM and 1874 cM, respectively. The average marker densities of these two maps were one SNP in 1.23–1.25 cM. A total of 1114 shared SNP markers were used to merge the two component maps. An integrated linkage map consisted of 2321 markers and spanned the cumulative length of 2052 cM. The composite map showed a substantial improvement in marker density, with one SNP marker in every 0.89 cM. To our knowledge, this is the most saturated genetic map in rubber tree to date. This integrated map allowed us to anchor 28,965 contigs, covering 135 Mb or 12% of the published rubber tree genome. We demonstrated that GBS is a robust and cost-effective approach for generating a common set of genome-wide SNP data suitable for constructing integrated linkage maps from multiple populations in a highly heterozygous agricultural species.

## Introduction

Para rubber tree [*Hevea brasiliensis* (Willd.) Muell.-Arg.] is a monoecious, highly outcrossing species that belongs to the family Euphorbiceae. Of the 10 *Hevea* species, *H. brasiliensis* is the only species that produces commercially viable quantity of high-quality natural rubber, accounting for more than 98% of the total production worldwide (Priyadarshan and Gonçalves, 2003). Unfortunately, its heterozygous nature and long growing cycles (at least 5 years before latex collection) have slowed down the development of high-yielding cultivars through conventional breeding with recurrent selection schemes. The ability to identify and select for individuals possessing desirable traits at an early stage through marker-assisted selection will help save time and resources required to develop superior cultivars.

In order to facilitate quantitative trait loci (QTL) mapping, several research groups have constructed genetic linkage maps from both intraspecific and interspecific crosses. Lespinasse et al. (2000) developed the first rubber tree genetic map from an interspecific cross between *H. brasiliensis* and *H. benthamiana*. The map consisted of 717 marker loci, most of which were restriction fragment length polymorphisms (RFLPs) and amplified fragment length polymorphisms (AFLPs) (Lespinasse et al., 2000). More recently, microsatellite or simple sequence repeat (SSR) markers have been employed in the construction of linkage maps in this species (Le Guen et al., 2011; Triwitayakorn et al., 2011; Souza et al., 2013). However, the number of SSR markers placed on each map was relatively small, ranging between 97 (Triwitayakorn et al., 2011) and 284 (Souza et al., 2013), and the average distances between adjacent markers on those maps were fairly large (between 8 and 11.9 cM), rendering them less practical for any downstream marker-trait association analyses.

To achieve high-density linkage maps, researchers have shifted from anonymous markers such as AFLPs and microsatellites to direct analyses of sequence variations, including single nucleotide polymorphisms (SNPs) (Zhang et al., 2013; Huang et al., 2014; Pootakham et al., 2014). SNPs are abundant in plant genomes, and their usefulness as genetic markers have been well established over the past decade. Recent studies of sequence diversities have shown that SNP frequencies in plants are one in every 100–300 bp (Edwards et al., 2007). Their ubiquity makes them valuable for genetic mapping, particularly for the generation of high-density linkage maps. Advances in high-throughput, next generation sequencing technologies have enabled initial efforts in SNP discovery in rubber tree (Pootakham et al., 2011; Mantello et al., 2014). Recently, next generation sequencing has been coupled with genome complexity reduction techniques and barcoding to identify and genotype a set of common SNPs in a mapping population. This strategy is referred to as genotyping-by-sequencing (GBS) (Elshire et al., 2011). GBS utilizes restriction endonucleases to digest the genome into fragments, which are subsequently sequenced on high-throughput platforms. Low cost per sample in GBS can be achieved by multiplexing samples from many individuals using unique DNA barcodes, which are ligated to digested fragments prior to sequencing (Elshire et al., 2011). GBS has successfully been used to generate high-density linkage maps in several organisms, including non-model species (Ward et al., 2013; He et al., 2014; Huang et al., 2014).

Consensus mapping has a potential to overcome the limitations typical of mapping information derived from single populations, especially the presence of regions with low marker density or lack of polymorphisms due to identity by descent (Maccaferri et al., 2007). Typically, a set of common markers present among individual maps are used as anchor points to integrate multiple maps and establish a consensus linkage map (Yan et al., 2005; N'Diaye et al., 2008). The use of multiple populations to obtain a consensus map provides greater coverage of the genome since it is unlikely that multiple parental lines would be monomorphic in the same regions. An increase in overall population size from combining multiple populations also improves the chances of capturing more recombination events. A consensus map allows for comparison and localization of markers that do not segregate in one population with those in another population. Additionally, it facilitates direct comparisons of QTL positions identified in various genetic backgrounds, enabling the development of polymorphic markers for marker-assisted breeding in diverse populations (Stam, 1993; N'Diaye et al., 2008).

In this study, we employed GBS technique to perform a genome-wide SNP discovery and genotyping of two rubber tree mapping populations. We generated single-population linkage maps and used common SNP markers as bridges to merge them into a high-density integrated genetic map. To our knowledge, this is the first attempt to apply GBS technique to identify SNP markers and construct high-density SNP-based linkage maps in rubber tree. SNP markers reported here will expand the existing repertoire of available molecular markers in *H. brasiliensis*, and the integrated genetic map presented will be useful for future breeding programs, association studies with desirable agronomic traits, genetic diversity analyses and phylogenetic studies.

## Materials and methods

### Plant materials

We used two rubber tree mapping populations in this study. Population P consisted of 118 F_1_ progeny derived from a cross pollination between a female parent BPM24 and a male parent RRIM600, while population C consisted of 79 F_1_ progeny derived from a cross pollination between a female parent BPM24 and a male parent RRIC110. The female parent of both crosses, BPM24, is a descendent of a GT1 × AVROS1734 cross. RRIM600, a widely cultivated accession, was obtained from a cross between Tjir 1 and PB86 whereas RRIC110 was a derived from a RRIC7 × LCB1320 cross. Both populations are being maintained at the Rubber Research Institute of Thailand, Ministry of Agriculture and Cooperatives, Thailand. Young leaf samples were collected, immediately frozen in liquid nitrogen and preserved at −80° until DNA extraction. DNA was isolated using a DNeasy Plant Mini Kit (Qiagen, Carlsbad, CA, USA), and the quality was checked on agarose gel to ensure that the samples were not degraded or contaminated with ribosomal RNA. DNA quantity was assessed by the NanoDrop ND-1000 Spectrophotometer, and the samples were diluted to 20 ng/μL for library construction.

### GBS library construction and ion proton sequencing

To prepare the reduced representation libraries for sequencing, we followed the GBS protocol using two enzymes (*Pst*I/*Msp*I) and a Y-adapter by Mascher et al. (2013) with a slight modification mentioned below. Two sets of adapters compatible with the primers of Ion Torrent Proton sequencing platform were synthesized by IDT (Singapore). To enable multiplex sequencing of the libraries, the forward adapters contained 9-bp unique barcodes in addition to the Ion Forward adapter and a *Pst*I restriction site. The reverse adapter (Y-adapter) contained the Ion reverse priming site and was designed such that amplification of the more common *Msp*I*-Msp*I fragments was prevented (Mascher et al., 2013).

The digestion of genomic DNA and the adapter ligation were performed as described in Mascher et al. (2013). We examined the size distribution of the final PCR-amplified library fragments using the BioAnalyzer 2100 (Agilent Technologies, Santa Clara, CA, USA) and noticed the prevalence of products between 100 and 200 bp. In order to take full advantage of the Ion PI™ Template OT2 200 Kit (Life Technologies, Grand Island, NY, USA), which supports 200-base read libraries, we modified the protocol by size-selecting fragments of ~270 bp (combined length of the forward and reverse adapter sequences was ~70 bp) using the E-Gel® SizeSelect™ Agarose Gels (Life Technologies, Grand Island, NY, USA). The libraries were subsequently quantified using the 2100 Bioanalyzer High Sensitivity DNA kit (Agilent Technologies, Santa Clara, CA, USA) and 8 μL of the diluted libraries (at 100 pM) were used as templates for emulsion PCR amplification. We multiplexed between 14 and 16 samples per run. The libraries were sequenced on the Ion Proton PI™ Chips according to the manufacturer's protocol (Life Technologies, Grand Island, NY, USA).

### Sequence data analysis and SNP genotype calling

Raw reads were de-multiplexed according to the barcodes and the adapter/barcode sequences were trimmed using the standard Ion Torrent™ Suite Software. Sequence data were submitted to the National Center for Biotechnology Information Short Read Archive (SRP057430). Clean reads were aligned to the rubber tree reference genome (Rahman et al., 2013) using the Ion Torrent™ Suite Software Alignment Plugin [Torrent Mapping Alignment Program (TMAP) Version 4.0.6] and the variants were called using the Ion Torrent VariantCaller (GATK v1.4-749-g8b996e2; Life Technologies, Grand Island, NY, USA). The parameters used for TMAP were as follows: number of bases to increase the seed for each seed increase iteration – 8, minimum seed length – −1, maximum seed length – 48, and maximum interval size to accept a hit – 20. Raw reads that could be mapped to multiple locations were filtered out and were not used for SNP calling. The following (default) parameter setting was applied for GATK: minimum sequence match on both sides of the variants – 5, minimum support for a variant to be evaluated – 6, minimum frequency of the variant to be reported – 0.15, and maximum relative strand bias – 0.8. SNPEff software was used to analyze effects of the mutations in coding sequences (Cingolani et al., 2012), with the rubber tree genome reference (Rahman et al., 2013) and the general feature format (GFF) annotation input files (Shearman et al., 2015).

### Construction of linkage maps from single populations

Linkage maps were constructed independently for each mapping population. Loci that were completely linked (i.e., displayed identical segregation pattern) were excluded from the data set before the marker order within the groups was determined. Polymorphic markers were classified into two categories according to their segregation patterns. The test-cross markers (AA × AB/BB × AB or AB × AA/AB × BB) segregated in a 1:1 ratio while the inter-cross markers (AB × AB) segregated in a 1:2:1 ratio. For each locus, we tested the pattern of allelic segregation for χ^2^ goodness-of-fit to expected Mendelian segregation ratios, and markers with significant segregation distortion (χ^2^ test *p*-value < 0.01) were excluded from further analysis. Non-segregating markers in AA × BB configuration were also discarded prior to map construction. Linkage analysis was performed with JoinMap v 3.0 (Van Ooijen and Voorrips, 2001) using the parameters set for the cross-pollinated (CP) population type. Initial assignment to linkage groups was based on the logarithm of the odds (LOD) threshold of 6.0 for each marker pair. We used linkages with a recombination rate (REC) < 0.4, a map LOD value of 0.05 and a goodness-of-fit jump threshold of five for inclusion into the map and for the calculation of the linear order of the markers within a linkage group. Recombination fractions between markers were converted to map distances in centiMorgans (cM) using the Kosambi mapping function (Kosambi, 1944). Linkage maps were drawn using Matplotlib package in Python (Hunter, 2007).

### Generation of the integrated map from individual populations

In order to use the merged map to anchor as many contigs from genome sequence as possible, redundant markers that had been removed prior to single-population map estimations were reincorporated into the component maps. The reincorporated markers were anchored to the linkage maps based on the positions of their corresponding representative markers. Homologous linkage groups from both genetic maps were identified according to shared markers and were merged using JoinMap v 3.0, which combined segregation data from individual maps and calculated consensus maps based on the assumption of homogeneous recombination rates across mapping populations (Van Ooijen and Voorrips, 2001). Common markers on homologous linkage groups from the component maps served as bridges to integrate them into a single consensus map. In one case where a linkage group from population P (LG4) corresponded to two linkage groups from population C (LG12 and LG13), LG4 was integrated with LG12 first and the merged linkage group was subsequently combined with LG13. The composite map consisted of all shared markers plus unique markers (specific to each population) and included estimated distances between marker loci in Kosambi cM.

## Results

### SNP discovery in rubber tree using genotyping-by-sequencing

To identify single nucleotide variations present in the populations used for map estimation, we generated *Pst*I-*Msp*I reduced representation libraries from genomic DNA of the parental varieties. To ensure sufficient read depths at potential SNP loci, we performed deeper sequencing of parental lines, obtaining a total of 30,906,485 raw reads covering 4.36 Gb of sequence data (12,246,755, 9,231,329 and 9,428,401 raw reads for RRIM600, BPM24 and RRIC110, respectively). Cleaned reads with quality scores of ≥ 20 were mapped against the publicly available rubber tree genome assembly with GATK (McKenna et al., 2010). The GBS reads aligned to 169,454 contigs from the published reference genome, 1066 of which overlapped with contigs that had previously been anchored to linkage maps (Rahman et al., 2013). Total mapped regions covered by ~200-bp fragments from *Pst*I-*Msp*I libraries were approximately 9.3 Mb, representing 0.84% of the published genome sequence.

Relative to the reference sequences, we identified a total of 21,353 single nucleotide substitutions (Table 1; a complete list of all SNPs identified in this study and their positions is provided in Data Sheet 1). The frequency of SNPs discovered using *Pst*I-*Msp*I GBS libraries was one in every 308 nucleotides. The majority of the nucleotide variations detected (55.43%) were transitions (A↔G or C↔T), whereas transversion events (A↔C, A↔T, C↔G, or G↔T) accounted for 44.57% (Table 1). The transitions between C↔T and A↔G appeared to be the most prevalent, with each representing approximately 27.7% of the total polymorphisms. On the other hand, C↔G transversion was the least common type of change, representing merely 9.23% of total polymorphisms.

**Table 1:** **Summary of single nucleotide substitutions identified in rubber tree**.

**Total SNPs**	**21,353**	**100 (%)**
**Transitions:**	**(11,836)**	**(55.43)**
A/G	5917	27.71
C/T	5919	27.72
**Transversions:**	**(9517)**	**(44.57)**
A/C	2462	11.53
A/T	2737	12.82
C/G	1971	9.23
G/T	2347	10.99

### Analysis of synonymous and non-synonymous SNPs in rubber tree coding regions

We were able to categorize the effect of 17,251 SNPs using the SNPEff software (Cingolani et al., 2012). The other 4102 SNPs were excluded from the analysis since they were not located within the annotated sequences in the input GFF file. The majority of the single nucleotide substitutions (41.4%) identified using GBS approach was distributed in exons, while the polymorphisms in introns represented 12.3% of all the substitutions analyzed. Approximately, one third of the variants examined were located in intergenic regions, and the remaining SNPs occurred in the regions within 5 kb immediately upstream or downstream of coding sequences. We also employed the SNPEff software to identify nucleotide substitutions that resulted in changes in amino acid (i.e., non-synonymous) and those that did not (i.e., synonymous). Among 7139 biallelic SNPs identified within the exonic regions, 35.6% were synonymous (silent) mutations, and the majority of non-synonymous substitutions were non-conservative mutations (Table S1).

### SNP genotyping of the mapping populations and informative markers identified

Genomic DNA from 197 individuals (118 F_1_ progeny from population P and 79 F_1_ progeny from population C) were used to prepare reduced representation libraries, each multiplexing between 14 and 16 lines, and sequenced on Ion Proton PI™ chips. A total of 1,003,904,519 raw reads, covering 135 Gb of sequence data were obtained from both populations, with an average of 5,095,962 reads per sample and a mean read length of 133 bases (Data Sheet 1). Approximately 81% of the total bases had a quality score of 20 or better, and 89.5% of the barcode/adapter-trimmed reads were able to align to the reference genome (Data Sheet 1).

A total of 7345 and 6678 SNP markers were called from populations P and C, respectively, with fewer than 50% missing data using the default parameter settings (read depth ≥ 6) on the Ion Torrent VariantCaller (see Materials and Methods). Missing data inherent in GBS along with genotyping errors often lead to inaccurate ordering of markers during map estimation. In order to minimize errors stemming from these factors, we were stringent in selecting SNP markers and included only genotypes with quality scores ≥ 20 and read depths ≥ 30 with fewer than 10% missing data in the construction of linkage maps.

Due to a long life cycle and inbreeding depression, it is difficult to obtain homozygous inbred lines in rubber tree. The conventional approaches of using F_2_ and backcross populations to generate linkage maps cannot be applied to this species. In perennial plants, genetic maps are typically constructed from a full-sib cross or F_1_ population using a double pseudo-testcross strategy. For bi-allelic markers such as SNPs, three marker configurations are considered informative for the calculation of the map: the inter-cross configuration where both parents are heterozygous (AB × AB) and two testcross configurations where one parent is heterozygous while the other parent is homozygous (AB × AA/AB × BB or AA × AB/BB × AB). Only SNP markers that followed these segregation patterns were retained for map construction.

After applying aforementioned criteria to filter the markers, a total of 2995 segregating SNPs were identified in population P and of those, 1223 markers were heterozygous in BPM24, and 969 markers were in the heterozygous in RRIM600. The remaining 803 markers were heterozygous in both parents. For population C, a set of 3124 segregating markers was identified: 1195 markers were heterozygous in BPM24, 1118 markers were heterozygous in RRIC110 and finally 811 markers were heterozygous in both lines. The average read depth of the 2995 SNP loci in each individual from population P was 199 ± 40, while the average read depth of the 3124 SNP positions in each sample from population C was 196 ± 47.

### Map construction from single populations

Chi-square analyses were performed on both sets of 2995 and 3124 informative markers from populations P and C, respectively, to evaluate their conformity to the expected Mendelian segregation ratios. Additionally, markers with identical segregation patterns were also discarded, and final sets of 1696 and 1613 stringently selected markers were used for the linkage map construction of populations P and C, respectively.

**Figure 1:**
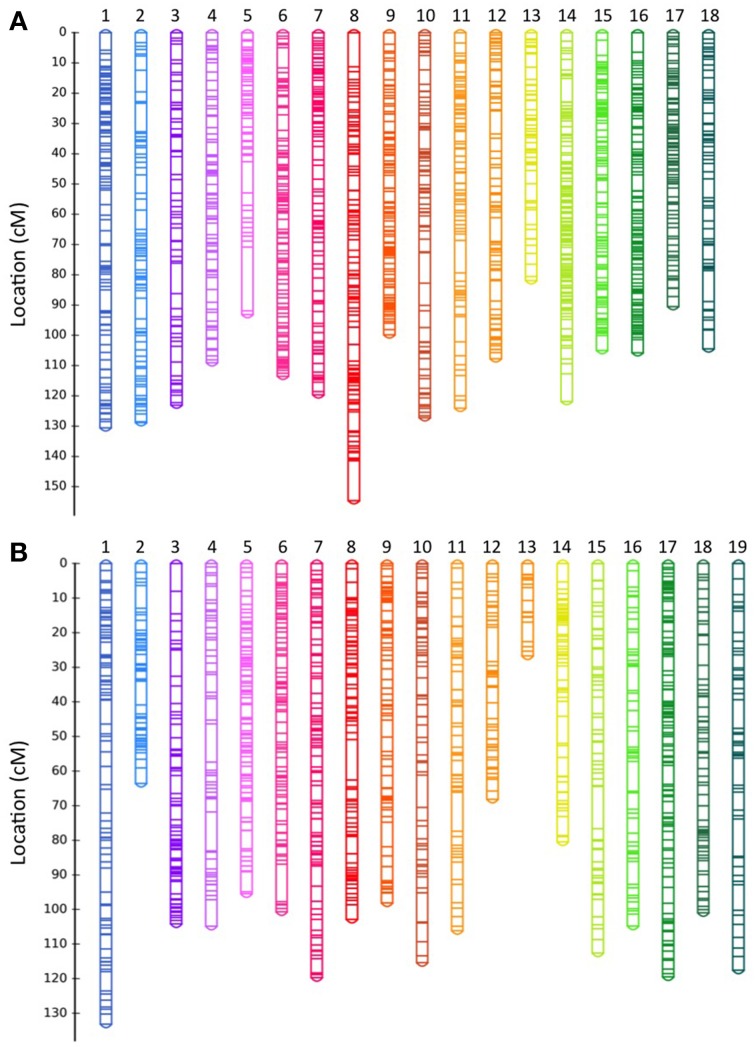
**GBS-based genetic linkage maps of rubber tree derived from F_1_ progeny from (A) BPM24 × RRIM600 and (B) BPM24 × RRIC110 crosses**. Linkage group numbers (assigned arbitrarily by JoinMap) are indicated at the top of each map. Details of SNP markers located on the maps are included in Data Sheet 2.

The genetic map derived from BPM24 × RRIM600 cross (population P) comprised 1638 non-redundant SNP markers spread over 18 linkage groups, which corresponded to the reported haploid chromosome number in rubber tree (Figure 1A and Table 2) (Leitch et al., 1998). The map encompassed 2041 cM, with linkage groups ranging from 81.96 cM (LG13) to 154.63 cM (LG8). The number of unique markers mapped to each linkage group varied from 58 SNPs in LG13 to 141 SNPs in LG14, with a mean of 91 SNPs per linkage group (Table 2).

**Table 2:** **Distribution of SNP markers on the linkage map derived from population P (BPM24 × RRIM600)**.

**Linkage group**	**Number of markers**		**Length (cM)**	**Average marker interval (cM)**	**Maximum interval (cM)**
	**All SNPs**	**Non-redundant SNPs**			
LG 1	105	105	130.56	1.26	6.86
LG 2	77	75	128.85	1.74	9.42
LG 3	63	61	123.04	2.05	10.06
LG 4	80	78	109.08	1.42	4.24
LG 5	74	66	93.15	1.43	21.01
LG 6	123	121	113.52	0.95	6.70
LG 7	115	108	119.68	1.12	4.57
LG 8	110	106	154.63	1.47	13.20
LG 9	109	105	99.84	0.96	4.20
LG 10	87	82	127.22	1.57	10.00
LG 11	82	80	124.08	1.57	10.70
LG 12	74	71	107.77	1.54	7.88
LG 13	60	58	81.96	1.44	5.67
LG 14	146	141	121.85	0.87	9.16
LG 15	113	108	105.01	0.98	5.07
LG 16	126	116	105.75	0.92	6.42
LG 17	98	95	90.51	0.96	3.34
LG 18	62	62	104.92	1.71	10.20
Average	94.66	91.00	113.41	1.25	–
**Total**	**1704**	**1638**	**2041.42**	**–**	**–**

The genetic map of BPM24 × RRIC110 (population C) consisted of 1540 SNP non-redundant markers and spanned the cumulative length of 1874 cM (Figure 1B and Table 3). Linkage groups ranged from 26.72 cM (LG13) to 133.14 cM (LG1) in length, and the number of unique markers placed on each group varied from 21 SNPs in LG13 to 135 SNPs in LG17, with an average of 81 markers per linkage group (Table 3). There were a total of 971 non-redundant SNP markers shared between the two component maps, with 667 and 569 markers mapped exclusively to populations P and C, respectively (Figure S1). The average inter-marker distances of the two maps were almost identical, 1.25 cM from the BPM24 × RRIM600 map and 1.23 cM from the BPM24 **×** RRIC110 map (Tables 2, 3), with 67% of the intervals smaller than 1.23 cM.

**Table 3:** **Distribution of SNP markers on the linkage map derived from population C (BPM24 × RRIC110)**.

**Linkage group**	**Number of markers**		**Length (cM)**	**Average marker interval (cM)**	**Maximum interval (cM)**
	**All SNPs**	**Non-redundant SNPs**			
LG 1	109	100	133.14	1.34	6.94
LG 2	68	61	63.55	1.06	6.90
LG 3	104	95	104.18	1.11	8.04
LG 4	66	61	104.85	1.75	11.20
LG 5	123	110	95.49	0.88	5.87
LG 6	130	110	100.69	0.92	10.90
LG 7	117	108	119.17	1.12	5.85
LG 8	117	94	102.85	1.11	11.70
LG 9	93	87	98.13	1.14	7.11
LG 10	94	82	115.37	1.42	9.33
LG 11	78	75	106.14	1.43	11.30
LG 12	62	53	68.13	1.31	9.77
LG 13	21	21	26.72	1.34	5.66
LG 14	87	73	80.46	1.12	7.81
LG 15	77	71	112.61	1.61	12.20
LG 16	66	64	104.81	1.66	8.59
LG 17	153	135	119.50	0.89	5.50
LG 18	85	79	100.97	1.29	5.96
LG 19	69	61	117.55	1.96	10.80
Average	90.47	81.05	98.65	1.23	–
**Total**	**1719**	**1540**	**1874.31**	**–**	**–**

### Integration of two component maps

**Figure 2:**
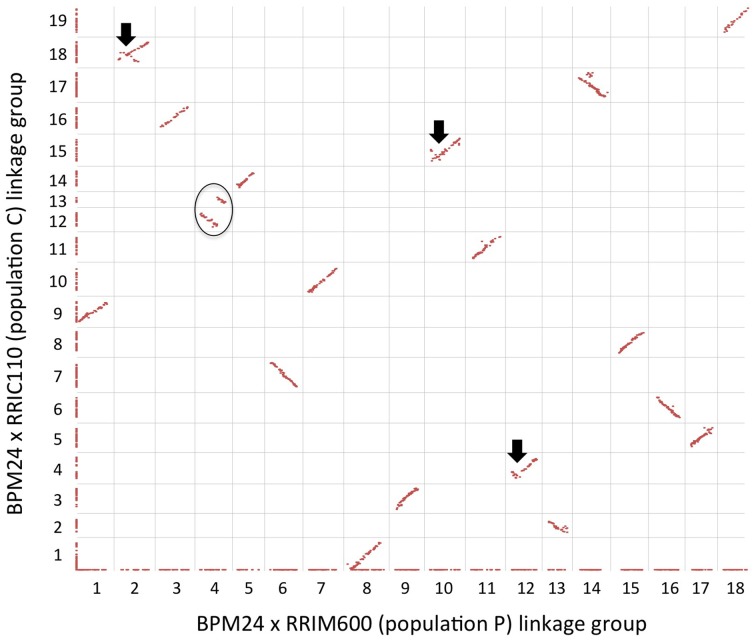
**Pairwise comparison between BPM24 × RRIM600 (population P) and BRM24 × RRIC110 (population C) linkage maps**. Shared markers are plotted according to their genetic positions on each map, whereas unique markers are plotted along the axes. A black circle indicates two linkage groups (LG12 and LG13) in population C that correspond to a single linkage group in population P (LG4). Black arrows indicate inconsistencies in marker order between the two maps.

Prior to constructing a composite map, we examined the order of shared markers between the two component maps using a scatter plot, and the majority of common markers exhibited long-ranged colinearity (Figure 2). The dot plot also revealed that both LG12 and LG13 in population C corresponded to LG4 in population P and should be joined into a single linkage group during map integration (Figure 2). Since we would like to anchor as many contigs as possible to the merged linkage map, we reintroduced genetically redundant markers (that had been excluded from previous map construction) into the component maps prior to the integration. To reduce the computational power required for map calculation, we reincorporated only redundant markers located on contigs that had not already been anchored to the single-population maps. Additional 66 and 179 SNP markers were reincorporated to linkage maps P and C, respectively, resulting in final genetic maps of 1704 markers (population P; Table 2) and 1719 markers (population C; Table 3). A total of 1114 common SNP markers on homologous linkage groups served as bridges to integrate the two component maps into a single consensus map with the expected 18 linkage groups (Figure S1).

**Figure 3:**
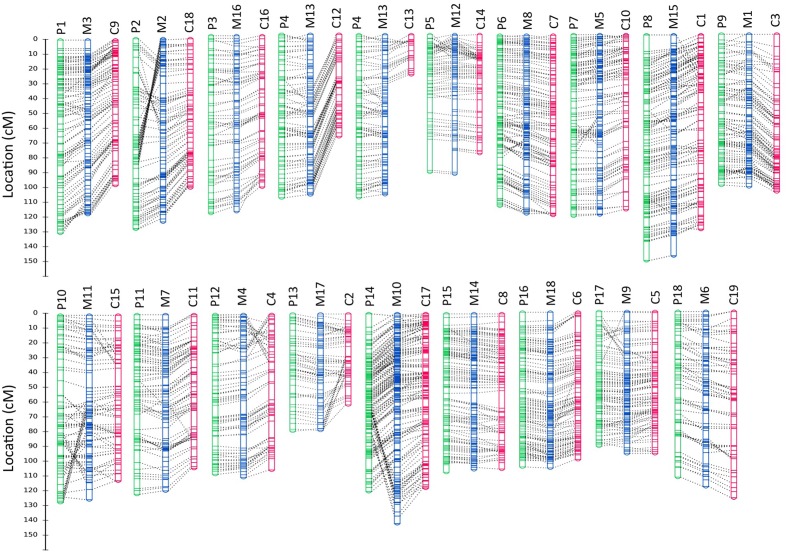
**Comparison of homologous linkage groups from the component and integrated genetic maps**. Linkage groups from BPM24 × RRIM600 (population P), BPM24 × RRIC110 (population C) and the merged map are displayed in green, magenta and blue, respectively. The genetic positions of SNP markers are shown on the left in Kosambi cM. Dotted lines connect homologous loci on each group, and linkage group numbers are indicated above the maps with P, C and M designating linkage groups from population P, population C and merged maps, respectively. Details of SNP markers located on the maps are included in Data Sheet 2.

The composite linkage map consisted of 2321 SNP markers (2105 with distinct genetic positions), including 1639 SNPs from population P and 1710 SNPs from population C (Figure 3 and Data Sheet 2). This genetic map spanned a cumulative length of 2052 cM, with the linkage groups ranging from 81.36 cM (LG17) to 151.31 cM (LG15; Table 4). The number of SNP markers placed on each linkage group varied from 84 SNPs in LG17 to 198 SNPs in LG10, with an average of 128 SNPs per linkage group. We compared our merged map to the published SSR-based map in Rahman et al. (2013) to identify common contigs in order to assign the linkage group numbers that were consistent with the literature. Detailed information on the consensus map, including genetic positions of SNP loci on the linkage groups and physical locations on the contigs/scaffolds is provided in Data Sheet 2.

**Table 4:** **Distribution of SNP markers on the integrated linkage map**.

**Linkage group**	**Number of markers**	**Length (cM)**	**Average marker interval (cM)**	**Maximum interval (cM)**
LG 1	143	95 (676)	100.88	0.71	4.74
LG 2	119	83 (742)	124.03	1.05	7.07
LG 3	140	71 (525)	117.87	0.84	5.77
LG 4	95	81 (652)	109.85	1.16	9.15
LG 5	139	77 (637)	118.82	0.86	4.32
LG 6	97	114 (853)	110.38	1.14	7.16
LG 7	103	108 (975)	121.72	1.19	7.53
LG 8	168	93 (564)	118.67	0.71	6.72
LG 9	146	96 (826)	95.55	0.65	5.85
LG 10	198	72 (440)	143.83	0.73	5.22
LG 11	113	76 (853)	125.40	1.11	10.24
LG 12	99	67 (559)	94.41	0.96	14.62
LG 13	111	63 (608)	107.00	0.97	6.55
LG 14	149	151 (1418)	103.22	0.69	6.67
LG 15	152	108 (806)	151.31	1.00	13.75
LG 16	96	112 (904)	121.78	1.28	4.49
LG 17	84	108 (954)	81.36	0.98	4.84
LG 18	169	69 (549)	106.49	0.63	5.67
Average	128.94	-	114.03	0.89	–
**Total**	**2321**	**1644 (13,541)**	**2052.57**	**–**	**–**

*^a^Rahman et al. (2013) Draft genome sequence of the rubber tree Hevea brasiliensis. BMC Genomics 14:75*.

The number of SNP markers with unique genetic position placed on the integrated map (2105) was substantially larger than those placed on the component maps (1638 on map P and 1540 on map C). The consensus map successfully bridged LG12 and LG13 from population C into a single linkage group. Additionally, the marker coverage across the genome was significantly improved since the integrated map allowed us to fill most of the large gaps on the individual maps (Figure 3 and Data Sheet 2). We observed only three gaps that were larger than 10 cM on the merged map (two at the terminal portions of LG12 and LG15 and one on LG11) while there were seven gaps larger than 10 cM on each of the component maps (Data Sheet 2).

### Anchoring sequenced contigs/scaffolds to the genetic map

The rubber tree genome assembly consisted of 608,017 scaffolds (1,223,364 contigs) covering 1119 Mb of sequences (Rahman et al., 2013). We were able to anchor 1644 SNP-containing contigs onto our integrated linkage map using 2321 GBS-derived markers. Additional number of contigs could be anchored by incorporating available scaffold data. A total of 13,541 contigs (representing 1423 scaffolds) were placed on the genetic map when scaffold information was taken into account (Data Sheet 2). When we further combined our map data with available contig placement information from the literature (Rahman et al., 2013), we were able anchor a total of 28,965 contigs (from 3875 scaffolds) onto the linkage map. The combined length of these contigs was 135 Mb, which was equivalent to 12% of the sequenced genome. The scaffold orientation on the map could not be determined since most of them were small (N50 length of 2972 bp) and only contained one marker. Nevertheless, this initial effort to anchor the sequenced contigs/scaffolds will be useful for future genome sequence assembly projects.

## Discussion

### Genome-wide SNP discovery using genotyping-by-sequencing

Rapid advances in high-throughput, next generation sequencing have revolutionized the approach in which SNP markers have recently been discovered and genotyped. Since genetic map construction can be accomplished with a small set of markers and does not require every single base to be sequenced, reduced representation methods are often coupled with available next generation sequencing technologies to further reduce the cost of SNP genotyping (Baird et al., 2008; Elshire et al., 2011). GBS is a rapid and efficient strategy that can simultaneously detect and score a large number of SNP markers, and this technique has successfully been employed to construct genetic linkage maps in several plant species (Poland et al., 2013; He et al., 2014). Here, we chose two methylation-sensitive restriction enzymes, *Pst*I and *Msp*I, for the construction of reduced representation libraries and selected digested products of ~200 bp for sequencing. Total mapped regions covered by those *Pst*I-*Msp*I fragments were approximately 9.3 Mb or 0.84% of the published genome sequence. The fraction of the rubber tree genome sequenced in *Pst*I-*Msp*I 200-bp libraries is comparable to the number reported in oil palm (14.2 Mb or 0.93% of the published genome sequence) when the same pair of enzymes were used to generate the libraries (Pootakham et al., 2015). If higher genome coverage is desired, reduced representation libraries can be prepared using restriction enzymes that recognize five bp, which will cut the genome more frequently than those with 6-base recognition sites. When *ApeK*I (a 5-cutter) was employed to reduce genome complexity in maize, the sequence tags obtained covered 51.8 Mb or 2.3% of the genome (Elshire et al., 2011).

We discovered a total of 21,353 SNPs from three rubber tree accessions (BPM24, RRIM600, and RRIC110) that are commonly used in breeding programs in Thailand. RRIM600 and RRIC110 are highly susceptible to Phytophthora and Corynespora, respectively, while BPM24 is resistant to both fungi (Churngchow and Rattarasarn, 2001; Thanseem et al., 2005). SNP markers developed from these genotypes will be useful for future mapping studies aiming to identify QTL that confer disease resistance in the BPM24 background. Based on the phylogenetic analysis, clone BPM24 is relatively distant from the other two cultivars (Nakkanong et al., 2008), thus some of the variants identified here may be rare and present only in the populations that share BPM24 genetic background.

The frequency of SNPs discovered using *Pst*I-*Msp*I GBS libraries was one in every 308 nucleotides, which was considerably higher than the previously reported frequency of one SNP in every 1.5 kb (Pootakham et al., 2011). We observed a transition:transversion ratio of 1.24, which was comparable to the previously published number (1.38) in rubber tree (Shearman et al., 2015). This ratio was also similar to the estimate reported for cassava (1.29), another member of the Euphorbiceae family (Pootakham et al., 2014). The bias in transition:transversion ratios commonly observed in SNP discovery probably reflects the frequent incidence of spontaneous deamination of 5-methylcytosine to thymine in the genome (Coulondre et al., 1978). The degeneracy of the genetic code and the selective pressure for gene conservation are likely accountable for the dominance of transitions over transversions. Synonymous substitutions are more often transitions than transversions, and there is a stronger selection against replacement substitutions, leading to higher occurrences of transitions (Moriyama and Powell, 1996). The percentage of non-synonymous substitutions found in rubber tree (64%) was comparable to the number reported in rice (56%) (Xu et al., 2012) but higher than the number observed in Arabidopsis (45%) (Clark et al., 2007).

Interestingly, a significant proportion of SNPs discovered in *Pst*I-*Msp*I reduced representation libraries were located either inside or in the vicinity (± 5 kb) of the genic regions. The observed enrichment in genic SNPs was likely the consequence of using two methylation-sensitive enzymes (*Pst*I and *Msp*I) to prepare the libraries. In plants, transposable and repetitive elements are heavily methylated while the euchromatic regions exhibit lower degrees of cytosine methylation (Zhang et al., 2010). Methylation-sensitive restriction enzymes have been employed in the construction of reduced representation libraries in order to enrich for hypomethylated gene space and avoid repetitive regions of the genome (Emberton et al., 2005; Nelson et al., 2008). *Pst*I and *Msp*I have been demonstrated to be efficient in reducing the number of clones with repeat elements. *Pst*I has also been shown to be effective in the enrichment of clones carrying the expressed portions of the genome when evaluated in maize and tobacco (Fellers, 2008).

### Construction of an integrated genetic linkage map

The use of falsely discovered SNPs for the construction of linkage maps could result in low-quality genetic maps. Since we would like to integrate these two maps into a single composite map, we applied stringent criteria to call and filter SNPs (quality scores ≥ 20 and read depths ≥ 30 with fewer than 10% missing data) to ensure that the single-population maps obtained were of high quality. The high proportions of input markers that were ultimately converted to genetic markers on the maps (1638/1696 for population P and 1540/1613 for population C) reflected the quality of these component maps. For each locus, we also tested the pattern of allelic segregation for χ^2^ goodness-of-fit to expected Mendelian segregation ratios, and markers with significant segregation distortion were excluded from further analysis. Even though the exclusion of distorted markers usually reduces the coverage of the genome, we decided to remove markers exhibiting significant deviation from the expected Mendelian ratio in order to obtain accurate genetic linkage maps. Segregation distortion frequently leads to a significant under- or overestimation of recombination fractions, which consequently influences the calculation of genetic distances between markers as well as the order of the markers (Lorieux et al., 1995).

We obtained genetic maps with 1638 and 1540 non-redundant markers spread over 18 and 19 linkage groups for Population P and C, respectively. The genetic map derived from population C had an excess of linkage groups (19 linkage groups; Figure 1B) relative to the haploid chromosome number (*n* = 18) (Leitch et al., 1998). Failure to achieve the basic chromosome number is likely due to a small population size (79 offspring in population C compared to 118 offspring in population P). Previous attempt to develop a genetic map from a population of similar size (81 F_1_ individuals) also resulted in a genetic map with excess number of linkage groups (Triwitayakorn et al., 2011). Most linkage groups had a one-to-one correspondence with a linkage group in the other population, with an exception of LG12 and LG13 from population C, which corresponded to a single linkage group (LG4) from population P (Figure 2).

The lengths of both maps (2041 cM for population P and 1874 cM for population C) were comparable to previously published linkage maps [2144 cM in Lespinasse et al. (2000) and 2441 cM in Le Guen et al. (2011)]. Our GBS-based linkage maps showed significantly higher marker densities compared to previously published microsatellite-based maps obtained from populations of similar or larger size [one marker every 3 cM in Lespinasse et al. (2000), one marker every 8 cM in Le Guen et al. (2011) and one marker every 10 cM in Souza et al. (2013)]. This is due primarily to a larger number of SNP markers placed on these genetic maps.

As a result of merging datasets from two populations, the average distance between adjacent markers across all 18 linkage groups on the integrated map (0.89 cM) was significantly smaller than the inter-marker distances of individual component maps, making this the most saturated genetic map in rubber tree to date (Table 4). Based on the estimated genome size of 2.15 Gb, the average genome-wide ratio of physical to genetic distance was 1047 Kb/cM, equivalent to one SNP marker per 1021 Kb. The average REC across all the linkage groups on the rubber tree consensus map is 0.95 cM/Mb, comparable to the rates reported for maize (0.75 cM/Mb) and sorghum (1.52 cM/Mb) (Henderson, 2012).

Except for few distal regions on the linkage groups, markers were distributed uniformly on both BPM24 × RRIM600 and BPM24 × RRIC110 maps. We observed seven gaps larger than 10 cM on each component map and between these two maps, the largest gap was 21 cM, located at the terminal portion of LG 5 on the BPM24 **×** RRIM600 map (Figure 1A). The presence of these gaps could result from the limitation of GBS-SNP markers in detecting polymorphisms in particular genome regions. Alternatively, they could represent recombination hotspots or sections of the genome that were identical-by-descent among the parental accessions and thus lack polymorphisms. Large gaps exhibiting low degree of polymorphisms have also been observed in genetic maps of other plant species, such as rye and common bean (Galeano et al., 2011; Milczarski et al., 2011).

A substantial number of common markers found between these two populations were expected because they shared BPM24 as a female parent. Occasionally, we observed local inconsistencies in the order of marker loci between the two single-population maps (Figure 2). These conflicts may be interpreted as technical errors from genotyping methods, or they may indicate the presence of local duplications or rearrangements in the parental genotypes (Han et al., 2011). These duplications may serve as hotspots of structural variations, significantly affecting the frequencies of recombination in different genetic backgrounds and thus resulting in inconsistent marker orders (Wang et al., 2011). Differences in recombination rates due to genomic structural variations between populations have been documented in other plant species, such as rapeseed (Lombard and Delourme, 2001) and maize (Beavis and Grant, 1991).

Despite local inversions (linkage groups P2 and M2, P10 and M11, Figure 3) or duplications (linkage groups P14 and M10, Figure 3) of neighboring markers, the SNP locus orders were mostly congruent between the component and the integrated maps. Minor discrepancies in marker positions are not uncommon in map integration (Hwang et al., 2009; Alheit et al., 2011). Such inconsistencies could reflect actual differences in genome organization between mapping populations or they could be attributed to either the effect of small sample size on the estimated gene orders or the differences in local recombination frequencies between populations. Rearrangements of closely linked markers, particularly those located at distal ends of linkage groups, have previously been observed in grapevine and cotton, among other plant species (Vezzulli et al., 2008; Xu et al., 2008).

Using the integrated linkage map obtained in this work, we were able to anchor 1644 SNP-containing contigs using 2321 markers. When the scaffold information was taken into account a total of 13,541 contigs from 1423 scaffolds were placed on the genetic map. Based on the previously reported draft genome sequence (Rahman et al., 2013), only 143 scaffolds covering 7.6 Mb have been placed into linkage groups. When we combined the information obtained from our integrated linkage map with available contig placement information, we were able to anchor a total of 28,965 contigs (from 3875 scaffolds) or 12% of the sequenced genome onto the linkage map. High-density genetic maps have proven to be extremely useful in genome sequence assembly projects, especially in species with highly fragmented assembly such as rubber tree (Ariyadasa et al., 2014).

In this study, we demonstrate that GBS is a rapid, efficient and inexpensive approach for constructing a high-density integrated linkage map. The use of several populations to obtain a consensus map provides increased genome coverage, as it is less likely for multiple accessions to all exhibit monomorphism in the same genomic regions. Additionally, an increase in overall population size (from combining multiple populations) improves the likelihoods of capturing more recombination events. Since GBS is a robust and highly reproducible technique, other laboratories will be able to construct linkage maps from different mapping populations based on the same set of SNP markers (by employing the same protocol for generating the reduced representation libraries). Given sufficient number of common markers among the linkage maps, their mapping data can be merged to ours and further improve the marker density of the final consensus map. The high-density integrated map reported here will serve as a valuable tool to the community for QTL analyses of important agronomic traits, marker-assisted breeding programs, comparative genome analysis and finally the assembly of a rubber tree whole-genome sequence.

## Author contributions

WP, STr, STa conceived and designed the experiments. WP, NJ, DS, TY, KT, KN, PR performed the experiments, including DNA extraction, library preparation and Ion Proton sequencing. PR, CS, STa performed SNP data analyses and linkage map construction. WP and STa wrote the manuscript. All authors read and approved the final version of the manuscript to be published.

### Conflict of interest statement

The authors declare that the research was conducted in the absence of any commercial or financial relationships that could be construed as a potential conflict of interest.

## Supplementary material

Figure S1 **Venn diagrams displaying the number of shared and unique markers on each map**. Map P was generated from F_1_ population of BPM24 × RRIM600 cross while map C was generated from F_1_ population of BPM24 × RRIC110 cross. **(A)** Number of non-redundant markers on maps P and C **(B)** number of all SNP markers after reincorporation of genetically redundant markers prior to map integration.
