# Supplementary figures and images for: Construction of a high-density integrated genetic linkage map of rubber tree (Hevea brasiliensis) using genotyping-by-sequencing (GBS)
Source: Front Plant Sci. 2015 May 27;6:367. doi: 10.3389/fpls.2015.00367 (PMC4444744; doi:10.3389/fpls.2015.00367)

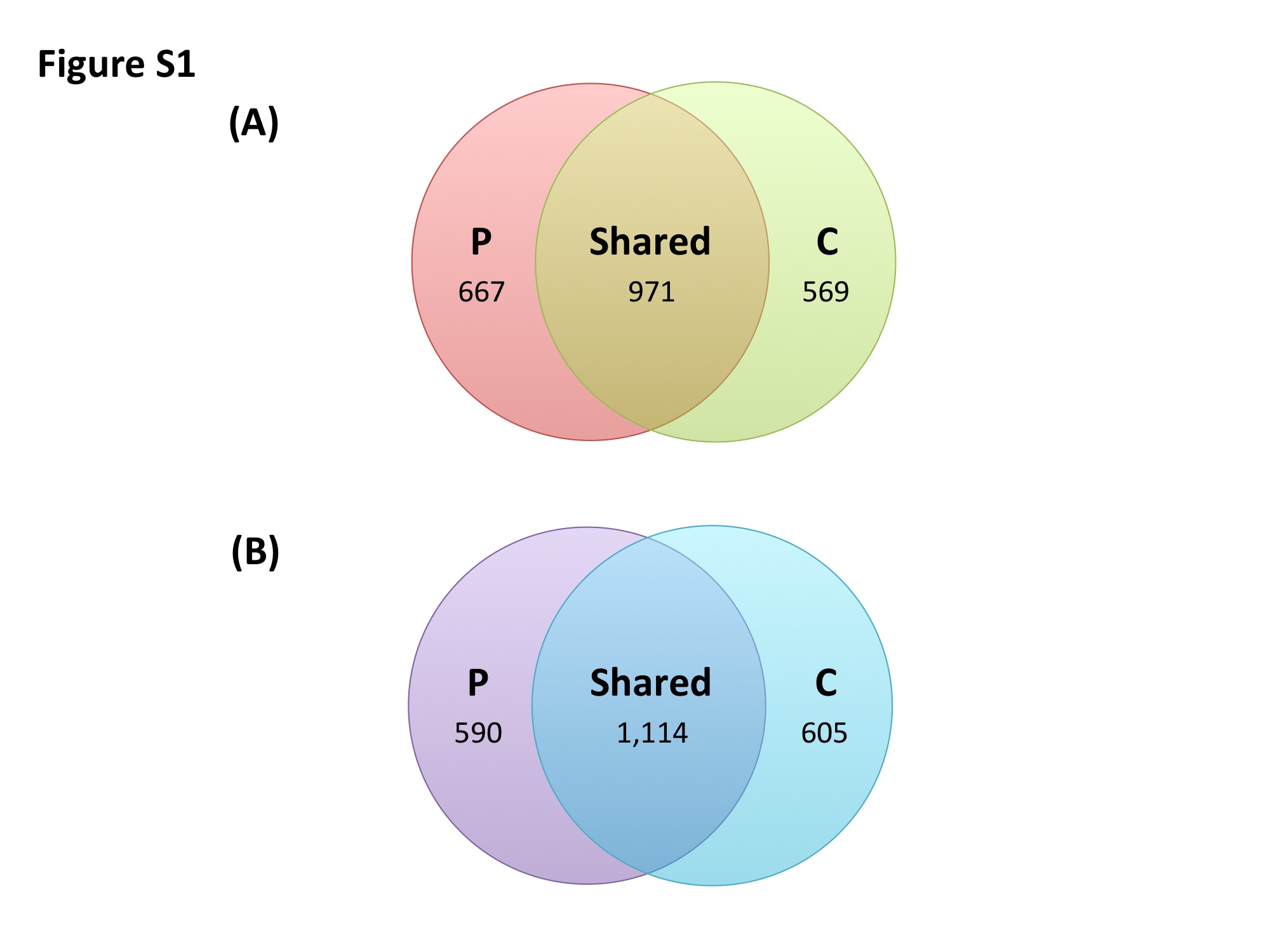

Supplement: Figure S1 — Venn diagrams displaying the number of shared and unique markers on each map. Map P was generated from F1 population of BPM24 × RRIM600 cross while map C was generated from F1 population of BPM24 × RRIC110 cross. (A) Number of non-redundant markers on maps P and C (B) number of all SNP markers after reincorporation of genetically redundant markers prior to map integration. [file Image1.JPEG]
