# Supplementary material for: Construction of a high-density integrated genetic linkage map of rubber tree (Hevea brasiliensis) using genotyping-by-sequencing (GBS)
Source: Front Plant Sci. 2015 May 27;6:367. doi: 10.3389/fpls.2015.00367 (PMC4444744; doi:10.3389/fpls.2015.00367)
Supplement: Supplementary file 2 [file Table1.DOCX]

**Table S1. Analysis of synonymous and non-synonymous changes in rubber tree SNPs.** Detailed information regarding the changes introduced by nucleotide substitutions.

| **Total number of exonic SNPs:** | **7,139** | **100%** |
| --- | --- | --- |
| **Synonymous mutations:**  **Non-synonymous mutations:**    Missense:  Conservative  Non-conservative  Nonsense:  Read-through: | 2,544  4,598  1,328  3,261  -  9 | 35.63%  64.40%  18.60%  45.68%  -  0.13% |
|  |  |  |
